# Pooled Analysis of Meningioma Risk Following Treatment for Childhood Cancer

**DOI:** 10.1001/jamaoncol.2022.4425

**Published:** 2022-10-06

**Authors:** Diana R. Withrow, Harald Anderson, Gregory T. Armstrong, Michael Hawkins, Neige Journy, Joseph P. Neglia, Florent de Vathaire, Margaret A. Tucker, Peter D. Inskip, Alina V. Brenner, Marilyn A. Stovall, Ibrahima Diallo, Amy Berrington de Gonzalez, Lene H. S. Veiga

**Affiliations:** 1 Nuffield Department of Primary Care Health Sciences, Medical Sciences Division, University of Oxford, Oxford, United Kingdom; 2 Representing the Nordic Countries Childhood Survival Group, Department of Cancer Epidemiology, Lund University, Lund, Sweden; 3 Epidemiology and Cancer Control Department, St Jude Children’s Research Hospital, Memphis, Tennessee; 4 Centre for Childhood Cancer Survivor Studies, Institute of Applied Health Research, University of Birmingham, The Robert Aitken Institute for Clinical Research Building, Birmingham, United Kingdom; 5 INSERM U1018, Centre for Research in Epidemiology and Population Health, Laboratory of Radiation Epidemiology & Cancer Survivorship Research, Paris-Saclay / Paris-Sud University, Gustave Roussy Cancer Campus, Villejuif, France; 6 Department of Pediatrics, Medical School, University of Minnesota, Minneapolis, Minnesota; 7 Division of Cancer Epidemiology and Genetics, National Cancer Institute, National Institutes of Health, Rockville, Maryland; 8 Radiation Effects Research Foundation, Hiroshima, Japan; 9 The University of Texas MD Anderson Cancer Center, Houston

## Abstract

**Importance:**

Meningioma is the most common subsequent neoplasm following cranial irradiation among survivors of childhood cancer, but there are still uncertainties regarding the magnitude of the radiation dose-response association, potential modifiers of radiation risks, and the role of chemotherapy.

**Objective:**

To evaluate meningioma risk in survivors of childhood cancer following radiotherapy and chemotherapy and identify possible modifying factors of radiation-associated risk.

**Design, Setting, and Participants:**

This international case-control study pooled data from 4 nested case-control studies of survivors of childhood cancer diagnosed between 1942 and 2000, followed through 2016. Cases were defined as participants diagnosed with a subsequent meningioma. Controls were matched to cases based on sex, age at first cancer diagnosis, and duration of follow-up. Data were analyzed from July 2019 to June 2022.

**Exposures:**

Radiation dose (Gy) to the meningioma site and cumulative chemotherapy doses, including intrathecal and systemic methotrexate doses.

**Main Outcomes and Measures:**

The main outcome was subsequent meningioma, assessed using odds ratios (ORs) and excess odds ratios per gray (EOR/Gy).

**Results:**

The analysis included 273 survivors of childhood cancer who developed meningioma (cases) and 738 survivors who did not (controls), with a total of 1011 individuals (median [IQR] age at first cancer diagnosis 5.0 [3.0-9.2] years; 599 [59.2%] female). Median (IQR) time since first cancer was 21.5 (15.0-27.0) years. Increasing radiation dose was associated with increased risk of meningioma (EOR/Gy, 1.44; 95% CI, 0.62-3.61), and there was no evidence of departure from linearity (*P* = .90). Compared with survivors who were not exposed to radiation therapy, those who received doses of 24 Gy or more had more than 30-fold higher odds of meningioma (OR, 33.66; 95% CI, 14.10-80.31). The radiation dose-response association was significantly lower among patients treated at age 10 years or older compared with those treated before age 10 years (EOR/Gy, 0.57; 95% CI, 0.18-1.91 vs 2.20; 95% CI, 0.87-6.31; *P* for heterogeneity = .03). Risk associated with radiation remained significantly elevated 30 years after exposure (EOR/Gy, 3.76; 95% CI, 0.77-29.15). We found an increased risk of meningioma among children who had received methotrexate (OR, 3.43; 95% CI, 1.56-7.57), but no evidence of a dose-response association or interaction with radiation dose.

**Conclusions and Relevance:**

These findings suggest that the meninges are highly radiosensitive, especially for children treated before age 10 years. These results support the reduction in whole-brain irradiation over recent decades and the prioritization of approaches that limit radiation exposure in healthy tissue for children. The persistence of elevated risks of meningiomas for 30 years after cranial radiotherapy could help inform surveillance guidelines.

## Key Points

**Question:**

What is the magnitude of the radiation dose-response association for meningioma, what is the role of chemotherapy, and do any factors modify the radiation risk?

**Findings:**

In this pooled analysis of 4 case-control studies including 1011 survivors of childhood cancer, risk of meningioma associated with radiation exposure increased with dose, risk was significantly higher among children exposed to radiation therapy before age 10 years, and the risk remained elevated for 30 years. Exposure to methotrexate was associated with increased risk of meningioma, but no dose-response association was observed.

**Meaning:**

These results support approaches that limit healthy tissue radiation exposure in children with cancer and could help inform surveillance guidelines for exposed patients.

## Teaser

This case-control study evaluates meningioma risk following radiotherapy and chemotherapy and identifies possible factors associated with modifying radiation-associated risk in survivors of childhood cancer.

## Introduction

Over the last 6 decades, childhood cancer survival has improved dramatically owing to advances in treatment.^1^ Therefore, attention has broadened to ensuring the long-term health of survivors of childhood cancer , including understanding the long-term outcomes associated with treatment, such as subsequent neoplasms (SNs).^2^ Meningioma is among the most common treatment-associated SNs diagnosed in survivors of childhood cancer, and the primary risk factor is cranial irradiation.^3^ Owing to a paucity of data, there are no recommendations for when to start surveillance for brain and central nervous system (CNS) tumors and whom to prioritize. Additional information about the patterns and magnitude of risks associated with treatment are needed.^4^

Results from several studies of survivors of childhood cancer suggest that the risk of subsequent brain tumors, including meningioma, increases with radiation dose. However, the magnitude of the meningioma risk varied considerably, with the estimated excess odds ratios per gray (EOR/Gy) ranging from 1.06 to 5.1. The ability to evaluate the shape of the dose-response association and its possible modification of by age at exposure, chemotherapy, and time since first cancer has been limited by small sample sizes.^5,6,7,8,9^ It has been suggested that methotrexate could contribute to meningioma risk, but it has been difficult to separate the outcomes associated with radiotherapy and chemotherapy.^5^ To address these limitations, we conducted a pooled analysis of 4 case-control studies of meningioma in survivors of childhood cancer with individual reconstructed radiation dose to the tumor location. We quantified the meningioma risk associated with radiation, investigated the shape of the dose-response association and potential factors that could modify this association, and evaluated the role of chemotherapy. The results can help inform meningioma screening guidelines for survivors of childhood cancer and contribute to our understanding of ionizing radiation and meningioma risk.

## Methods

For this pooled case-control study, all contributing studies underwent ethical approval, and informed consent of study participants occurred at host institutions. This pooled study used deidentified, secondary data, so further review and informed consent were waived by the host institutions (St Jude Children’s Research Hospital, Institut Gustave Roussy, the University of Birmingham, and Lund University). This study is reported following the Strengthening the Reporting of Observational Studies in Epidemiology (STROBE) reporting guideline.

### Study Populations

This pooled analysis included all 4 known nested case-control studies of subsequent brain and CNS tumors among survivors of childhood cancer that have individual dose estimates to the tumor location. These childhood cancer survivor studies were conducted in the United States and Canada,^6^ France,^7^ the United Kingdom,^5^ and Nordic countries.^9^ Individuals were treated for first primary cancers between 1942 and 2000. Cases were survivors of childhood cancer who developed a subsequent primary tumor of the brain or CNS, including meningioma (*International Classification of Diseases for Oncology, 3rd Edition* [*ICD-O-3*] codes: C70.0-C72.9). Controls were survivors of childhood cancer matched on age, sex, and duration of follow-up. The French and Nordic studies also matched on calendar year of first cancer.

The 4 studies are described in detail elsewhere and summarized in the eMethods and eTable 1 in the Supplement.^5,6,7,9^ Absorbed radiation doses to the meningioma location in the cases, and the corresponding locations in matched controls, were estimated based on individual recorded treatment parameters and phantom measurements. Detailed chemotherapy information for treatment of the first cancers was abstracted from medical records according to a standardized protocol.

### Organization of Data

Topography and histology codes were used to classify first and SNs types uniformly across studies. Meningioma was classified based on *ICD-O-3* histology codes 9530 to 9539. First cancers were classified using the Birch and Marsden scheme.^10^ Chemotherapy agents were grouped into major classes: alkylating agents, anthracyclines, epipodophyllotoxins, platinum-based compounds, and antimetabolites (intrathecal and systemic methotrexate). For all other variables, data sets were harmonized by applying a standard data dictionary to raw variables.

### Statistical Analysis

To estimate odds ratios (ORs) and 95% CIs for meningioma by first cancer type, exposure to chemotherapy (overall and by classes, yes or no), radiation (overall yes or no and dose quintiles), we used multivariable conditional logistic regression. We fitted models that allowed risk to increase with increasing radiation dose in a linear, quadratic, or linear-quadratic fashion, together with a negative log-linear or log-quadratic term to allow for the possible downturn in the radiation dose-response association due to cell-killing at high doses. The EOR model has the general form:

*EOR*(*d*) = (β_1_*^d^* + β_2_*d*^2^) × *exp*(β_3_*d* + β_4_*d*^2^)

where *EOR*(*d*) represents the EOR (OR− 1) as a function of radiation dose *d* with β_1_ through β_4_ as regression coefficients. The effect estimate is expressed per gray *(*EOR/Gy)*.* Likelihood ratio tests and the Akaike Information Criterion were used to determine the most parsimonious, best-fit model.

Radiation dose-response models were conditioned on matching factors from the original studies (including sex, age at first cancer diagnosis, and duration of follow-up [eTable 1 in the Supplement]) and adjusted for first cancer type (CNS, leukemia, others) and methotrexate receipt (yes or no). We did not adjust for study because we preserved the matching sets from each contributing study, adjusting the analysis intrinsically for study. Adjustment factors were selected based on association with meningioma in the multivariable analysis (*P* < .05). An influence analysis was conducted by omitting each first cancer type and each study, 1 at a time.

Since the British study^5^ had previously reported an association between meningioma and intrathecal methotrexate, we examined the risk associated with methotrexate (yes or no) and cumulative doses (milligrams per meters squared) by route of administration (intrathecal and systemic). We also evaluated the risk associated with an overall methotrexate score based on tertiles of dose of systemic and intrathecal methotrexate combined. Each study participant was assigned a score of 0, 1, 2, or 3 for each route of administration, depending on whether the participant received none or fell into the lower, middle, or upper third of the distribution, respectively. The scores of the intrathecal and systemic methotrexate were then summed for each study subject to obtain a methotrexate score, which ranged from 0 to 6. Participants with missing intrathecal or systemic methotrexate dose were excluded from this analysis.

We evaluated modification of the radiation dose-response association by age at first cancer diagnosis, time since first cancer (as proxy for first radiation exposure), attained age, sex, first cancer type, calendar year of follow-up, and classes of chemotherapy by including those variables in the exponential term and evaluating the goodness of fit by likelihood ratio tests. Statistical tests were 2-sided and based on α = .05. Data analysis was conducted from July 2019 to June 2022. All analyses were conducted using the PECAN module of Epicure, version 2.00.02 (Risk Sciences International).

## Results

### Study Participants

This study included a total of 1011 individuals (median [IQR] age at first diagnosis, 5.0 [3.0-9.2] years; 599 [59.2%] female), with 273 meningioma cases and 738 controls (Table 1). Central nervous system tumors were the most common first cancers among cases (153 individuals [56.0%]), whereas controls most commonly had leukemia (116 individuals [15.7%]), CNS (111 individuals [15.0%]), or Wilms tumors (125 individuals [16.9%]). Owing to matching, median (IQR) age at first cancer diagnosis (5.5 [3.0-9.3] years vs 5.0 [2.0-9.1] years) and time since first cancer diagnosis (21.5 [15.1-27.0] years vs 21.5 [15.0-27.0] years) were similar in cases and controls (Table 1). The median (IQR) age at meningioma diagnosis was 27.8 (21.3-34.0) years.

**Table 1.** Patient Characteristics

Characteristic	No. (%)	
	Cases (n = 273)	Controls (n = 738)
First cancer		
Leukemia	68 (24.9)	116 (15.7)
CNS cancer	153 (56.0)	111 (15.0)
Hodgkin lymphoma	9 (3.3)	55 (7.5)
Non-Hodgkin lymphoma	9 (3.3)	37 (5.0)
Kidney (Wilms tumor)	3 (1.1)	125 (16.9)
Neuroblastoma	4 (1.5)	71 (9.6)
Soft tissue sarcoma	6 (2.2)	73 (9.9)
Bone cancer	2 (0.7)	53 (7.2)
Retinoblastoma	5 (1.8)	23 (3.1)
Other	14 (5.1)	74 (10.0)
Sex		
Male	114 (41.8)	298 (40.4)
Female	159 (58.2)	440 (59.6)
Age at first cancer diagnosis, y		
0-4	117 (42.9)	338 (45.8)
5-9	94 (34.4)	220 (29.8)
10-14	47 (17.2)	127 (17.2)
15-20	15 (5.5)	53 (7.2)
Median (IQR)	5.5 (3.0-9.1)	5.0 (2.0-9.1)
Year of first cancer diagnosis		
1942-1959	16 (5.9)	31 (4.2)
1960-1969	43 (15.8)	97 (13.1)
1970-1979	140 (51.3)	391 (53.0)
1980-2000	47 (27.1)	219 (29.7)
Radiotherapy		
No	11 (4.0)	229 (31.0)
Yes	262 (96.0)	509 (69.0)
Chemotherapy^a^		
No	113 (41.4)	218 (29.5)
Yes	139 (50.9)	486 (65.9)
Unknown	21 (7.7)	34 (4.6)
Chemotherapy agents		
Alkylating agents	90 (33.0)	305 (41.3)
Anthracyclines	34 (12.5)	193 (26.1)
Epipodophyllotoxins	18 (6.6)	35 (4.7)
Platinum-based compounds	20 (7.3)	41 (5.6)
Antimetabolites	85 (31.1)	165 (22.4)
IT methotrexate	67 (24.5)	110 (14.9)
Systemic methotrexate	52 (19.0)	91 (12.3)
Time since first cancer, y		
0-4	2 (0.7)	6 (0.8)
5-14	60 (22.0)	162 (22.0)
15-24	119 (43.6)	322 (43.6)
≥25	92 (33.7)	248 (33.6)
Median (IQR)	21.5 (15.1-27.0)	21.5 (15.0-27.0)
Attained age, y		
<30	163 (59.7)	440 (59.6)
30-39	80 (29.3)	223 (30.2)
≥40	30 (11.0)	75 (10.2)
Median (IQR)	27.8 (21.3-34.0)	27.8 (20.2-34.0)
End of follow-up/y of meningioma diagnosis		
1951-1979	12 (4.4)	30 (4.1)
1980-1989	37 (13.6)	126 (17.1)
1990-1999	126 (46.2)	266 (36.0)
2000-2009	72 (26.4)	238 (32.3)
2010-2016	26 (9.5)	78 (10.6)

Abbreviations: CNS, central nervous system; IT, intrathecal.

^a^ May not add to total due to participants with unknown treatment status.

A higher proportion of cases than controls received radiotherapy (262 individuals [96.0%] vs 509 individuals [69.0%]), whereas chemotherapy was less common in cases than controls (139 individuals [50.9%] vs 486 individuals [65.9%]). A higher proportion of cases than controls received intrathecal (67 individuals [24.5%] vs 110 individuals [14.9%]) or systemic (52 individuals [19.0%] vs 91 individuals [12.3%]) methotrexate.

Methotrexate was most common among survivors of leukemia, non-Hodgkin lymphoma, and bone cancer (eTable 2 in the Supplement). Patients with leukemia more often received intrathecal methotrexate, whereas bone cancer patients more often received systemic methotrexate. Methotrexate was most common after 1970, with decreasing use of intrathecal and increasing use of systemic methotrexate after 1980. Patients who had radiation doses exceeding 20 Gy more often received methotrexate than those with lower radiation doses (eTable 2 in the Supplement). Among controls, radiotherapy dose was associated with first cancer type (highest among those with leukemia or CNS tumors), but there was no association between dose and age or calendar year of cancer diagnosis (eTable 3 in the Supplement).

### Risk Factors for Meningioma

Survivors of CNS tumors had higher odds of subsequent meningioma compared with those with other first cancers (OR, 2.89; 95% CI, 1.52-5.51), whereas survivors of leukemia did not have higher odds of meningioma (OR, 0.84; 95% CI, 0.38-1.82) (Table 2). Any radiotherapy exposure (OR, 11.02; 95% CI, 4.96-24.50), and any methotrexate receipt (OR, 3.43; 95% CI, 1.56-7.57) were associated with significantly greater odds of meningioma. Risk was similar for systemic (OR, 2.37; 95% CI, 1.22-4.58) and intrathecal (OR, 2.90; 95% CI, 1.27-6.62) methotrexate (Table 2). There were no significant associations for chemotherapy overall, alkylating agents, anthracyclines, platinum-based compounds, or epipodophyllotoxins.

**Table 2.** Odds of Subsequent Meningiomas Following Treatment for Childhood Cancer by Selected Variables

Variable	No. (%)		OR (95% CI)^a^	*P* value
	Cases	Controls		
Type of first cancer				
Others	52 (19.1)	511 (69.2)	1 [Reference]	<.001
Leukemia	68 (24.9)	116 (15.7)	0.84 (0.38-1.82)	
CNS	153 (56.0)	111 (15.0)	2.89 (1.52-5.51)	
Radiation treatment				
No	11 (4.0)	229 (31.0)	1 [Reference]	<.001
Yes	262 (96.0)	509 (69.0)	11.02 (4.96-24.50)	
Chemotherapy^b^				
No	113 (41.4)	218 (29.5)	1 [Reference]	.18
Yes	139 (50.9)	486 (65.9)	1.50 (0.82-2.75)	
Alkylating agents^b^				
No	162 (59.3)	399 (54.1)	1 [Reference]	.69
Yes	90 (33.0)	305 (41.3)	0.89 (0.51-1.55)	
Anthracyclines^b^				
No	218 (80.0)	511 (69.2)	1 [Reference]	.65
Yes	34 (12.5)	193 (26.1)	0.85 (0.42-1.71)	
Methotrexate^b^				
Any				
No	172 (63.0)	548 (74.3)	1 [Reference]	.001
Yes	80 (29.3)	157 (21.3)	3.43 (1.56-7.57)	
Intrathecal^b^				
No	172 (63.0)	556 (75.3)	1 [Reference]	.009
Yes	67 (24.5)	110 (14.9)	2.90 (1.27-6.62)	
Systemic^b^				
No	187 (68.5)	575 (77.9)	1 [Reference]	.01
Yes	52 (19.1)	91 (12.3)	2.37 (1.22-4.58)	
Epipodophyllotoxins^b^				
No	234 (85.7)	670 (90.8)	1 [Reference]	.08
Yes	18 (6.6)	35 (4.7)	2.61 (0.88-7.72)	
Platinum-based compounds^b^				
No	232 (85.0)	664 (90.0)	1 [Reference]	.28
Yes	20 (7.3)	41 (51.6)	1.79 (0.61-5.27)	

Abbreviations: CNS, central nervous system; OR, odds ratio.

^a^ ORs were estimated using conditional logistic regression adjusted for matching variables, type of first cancer (leukemia, CNS cancer, other types), methotrexate (yes/no), and categorical radiation dose as appropriate.

^b^ May not add to total due to participants with unknown treatment status.

### Radiation Dose-Response Findings

**Figure 1.**
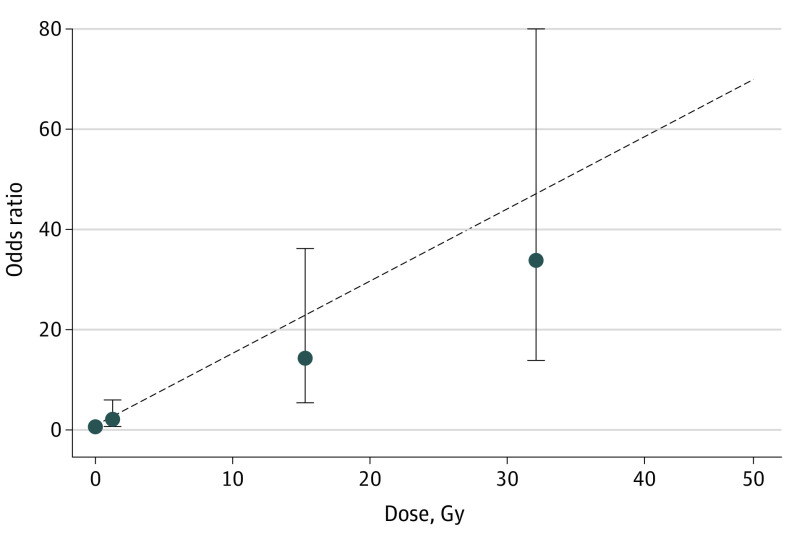
Odds of Subsequent Meningiomas by Categories of Radiation Dose and Fitted Linear Dose-Response Model Model included adjustment for matching variables, type of first cancer (leukemia, central nervous system cancer, and other cancers), and methotrexate (yes vs no). Error bars indicate 95% CIs.

A linear radiation dose-response model best fit the data, with no evidence of upward curvature at lower doses or downward curvature at higher doses (eTable 4 in the Supplement). Risk of meningioma increased with increasing radiation dose to the meningioma location (EOR/Gy, 1.44; 95% CI, 0.62-3.61; *P* for trend < .001) (Table 3 and Figure 1). Compared with survivors who were not exposed to radiation therapy, we estimated an OR of 14.44 (95% CI, 5.73-36.39) among those who had received 4 to 24 Gy and 33.66 (95% CI, 14.10-80.31) among those who received more than 24 Gy. There was no evidence of heterogeneity of the EOR/Gy across studies (*P* for heterogeneity = .84) (eTable 5 in the Supplement), and there was no major change in the dose-response association when any single study or first cancer type was omitted (eTable 6 and eTable 7 in the Supplement).

**Table 3.** Radiation and Methotrexate Dose-Response Association for Meningioma Among Survivors of Childhood Cancers

Variable	No. (%)		OR (95% CI)^a^	*P* for trend
	Cases	Controls		
Radiation dose (mean), Gy ^b^				
0	13 (4.8)	267 (36.2)	1 [Reference]	NA
>0 to <0.40 (0.14)	4 (1.5)	176 (23.8)	0.57 (0.17-1.94)	
0.40 to <3.96 (1.33)	14 (5.1)	91 (12.3)	2.33 (0.87-6.22)	
3.96 to <24.0 (15.95)	43 (15.8)	68 (9.2)	14.44 (5.73-36.39)	
24.0 to -80 (33.49)	191 (70)	110 (14.9)	33.66 (14.10-80.31)	
Unknown	8 (2.9)	26 (3.5)	14.12 (4.21-47.31)	
EOR/Gy^c^	265	712	1.44 (0.62-3.61)	<.001
Methotrexate score^d^				
0	172 (69.1)	548 (79.2)	1 [Reference]	.004^e^
1	10 (4.0)	25 (3.6)	2.79 (0.75-10.40)	
2	28 (11.2)	36 (5.2)	3.65 (1.38-9.63)	
3	26 (10.4)	57 (8.2)	3.68 (1.43-9.48)	
≥4	13 (5.2)	26 (4.0)	4.48 (1.48-13.61)	
Methotrexate score (restricted to patients exposed to RT)^d^				
0	165 (69.6)	348 (79.3)	1 [Reference]	.02^f^
1	8 (3.4)	14 (3.2)	0.83 (0.15-4.47)	
2	28 (11.8)	30 (6.8)	2.60 (0.95-7.09)	
3	23 (9.7)	36 (8.2)	2.13 (0.75-6.03)	
≥4	13 (5.5)	11 (2.5)	3.95 (1.15-13.52)	

Abbreviations: EOR/Gy, excess odds ratio per gray; NA, not applicable; OR, odd ratio; RT, radiation therapy.

^a^ ORs were estimated using conditional logistic regression adjusted for matching variables, type of first cancer (leukemia, central nervous system cancer, other cancers), methotrexate (yes/no), and categorical radiation dose as appropriate.

^b^ Quintiles based on radiation dose distribution among controls. First and second quintiles were collapsed due to a lack of cases in the first quintile.

^c^ Participants with unknown radiation dose were excluded.

^d^ Intrathecal and systemic methotrexate dose distributions among controls were divided into tertiles. Each participant was assigned a score of 0, 1, 2, or 3 for each route of administration, depending on whether the subject received none or fell into the lower, middle, or upper third of the distribution, respectively. The scores of the intrathecal and systemic methotrexate were then summed for each participant to obtain a methotrexate score, which ranged from 0 to 6. Participants with missing intrathecal or systemic methotrexate dose were excluded from this analysis. Totals do not sum to study totals due to participants with unknown methotrexate receipt.

^e^ *P* for trend among participants exposed to methotrexate = .47.

^f^ *P* for trend among participants exposed to methotrexate = .53.

### Modification of the Radiation Dose-Response Association

**Figure 2.**
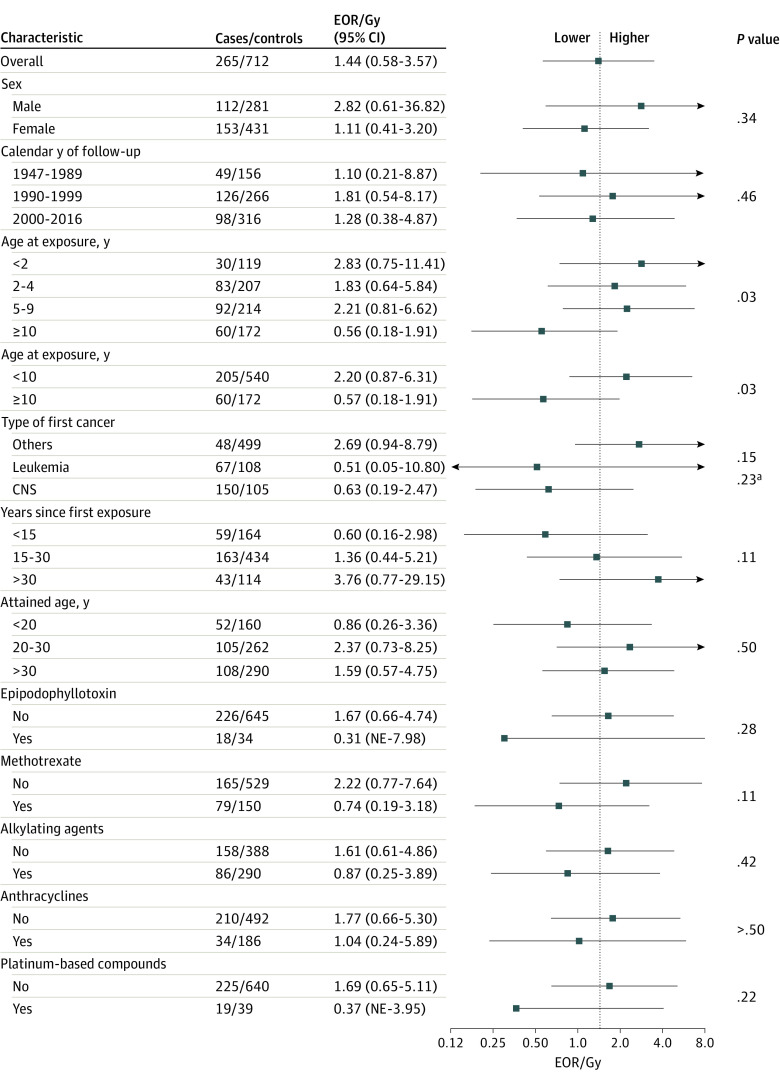
Estimates of Modification to the Linear Component of the Radiation Dose-Response Association Excess odds ratio (EOR) model included adjustment for type of first cancer (leukemia, central nervous system [CNS] cancer, other cancers) and methotrexate (yes vs no). Patients with missing radiation dose (8 cases, 26 controls) were excluded. For categorical variables, *P* values are for heterogeneity, whereas for variables that can be measured continuously, *P* for trend was used. NE indicates not estimated; error bars, 95% CI. ^a^Adjusted for effect modification by age at radiation exposure.

There was a significant trend of decreasing EOR/Gy with increasing age at exposure (individuals exposed at age <2 years: EOR/Gy, 2.83; 95% CI, 0.75-11.41; individuals exposed at age >10 years: EOR/Gy, 0.56; 95% CI, 0.18-1.91; *P* for trend = .03) (Figure 2). Survivors of childhood cancer treated before age 10 years exhibited higher risks associated with radiation compared with those treated at 10 years or older; however, there was no significant trend by age among those exposed at ages younger than 10. In exploratory analyses, we further evaluated modification of the dose-response association by age at exposure stratified by first cancer type (CNS vs non-CNS) and follow-up time (<20 vs ≥20 years) because of the strong correlation among these factors (eTable 8 in the Supplement). The higher EORs/Gy in patients treated at ages younger than 10 years persisted (eTable 9 and eTable 10 in the Supplement).

There was no significant difference in EOR/Gy for males (EOR/Gy, 2.82; 95% CI, 0.61-36.82) vs females (EOR/Gy, 1.11; 95% CI, 0.41-3.20; *P* = .34), nor for survivors of CNS tumors (EOR/Gy, 0.63; 95% CI, 0.19-2.47) or leukemia (EOR/Gy, 0.51; 95% CI, 0.05-10.80) compared with other first cancers (EOR/Gy, 2.69; 95% CI, 0.94-8.79; *P* = .15). Results remained unchanged with age at exposure included in the model as modifiers of the dose-response association. There was no significant change in the EOR/Gy with time since exposure, and a significant EOR/Gy was observed even at 30 years after exposure (EOR/Gy, 3.76; 95% CI, 0.77-29.15). There was no evidence of modification of the radiation dose-response association by attained age, calendar year of follow-up, or class of chemotherapeutic agent, including methotrexate (Figure 2).

### Chemotherapy Dose-Response Findings

Since methotrexate showed an association with meningioma risk, we explored a potential dose-response association. Using a score derived from tertiles of dose of intrathecal and systemic methotrexate combined, we found an overall significant increasing trend (*P* for trend = .004) but no trend among individuals exposed to methotrexate (*P* for trend = .47), suggesting the trend was driven by the nonexposed group (supporting data reported in Table 3). To consider residual confounding by radiation, we restricted to individuals exposed to radiation and found no significant dose-response association among those who had received any methotrexate (Table 3). Results remained unchanged in an influence analysis removing 1 study at a time (eTable 11 in the Supplement).

There was no dose-response association with either intrathecal (*P* for trend = .41; supporting data reported in eTable 12 in the Supplement) or systemic methotrexate dose (*P* for trend = .35; supporting data in eTable 12 in the Supplement). A trend of decreasing risk with increasing intrathecal methotrexate was observed when analyses were restricted to individuals with any intrathecal methotrexate exposure (exposed *P* for trend = .002; supporting data reported in eTable 12 in the Supplement). We also explored a potential dose-response association with epipodophyllotoxin receipt (OR, 2.61; 95%CI, 0.88-7.72) but found no association (eTable 13 in the Supplement).

## Discussion

This pooled case-control analysis constitutes the largest ever study of meningioma risk among survivors of childhood cancer, to our knowledge. Meningioma risk increased with radiation dose in a manner consistent with linearity. Patients who received doses of 24 Gy or more to the meningioma location had nearly 30-fold higher odds of meningioma compared with survivors of childhood cancer who did not receive radiation therapy. There was a higher EOR/Gy in individuals aged younger than 10 years compared with those aged 10 years and older at exposure. We found a 3-fold higher risk of meningioma after treatment with methotrexate but no evidence of a dose-response association among individuals exposed to methotrexate when a composite index of intrathecal and systemic methotrexate was used.

Cranial radiation exposure during childhood is an established risk factor for meningioma. While the nature of the case-control study prohibited us from estimating cumulative incidence, previous studies have estimated that survivors of childhood cancer exposed to any radiation have a cumulative incidence of meningioma of 0.3% at 15 years after diagnosis, and those exposed to cranial irradiation have a cumulative incidence of nearly 6% by age 40 years.^11,12^ To our knowledge, our results provide the most precise estimates of the dose-response association concerning meningioma risk following high-dose, fractionated radiation exposure. There are few comparable cohorts of children exposed to moderate to high doses of radiation. In survivors of the atomic bombs in Japan exposed to a wide range of radiation doses at high dose-rates, the excess relative risk per Gy (ERR/Gy) was estimated to be 2.05 (95% CI, 0.14-6.60) for children aged 0 to 10 years at exposure,^13^ which is consistent with our findings based on high-dose fractionated radiotherapy within the same age range (EOR/Gy, 2.20). This contrasts with results for most solid cancer sites, for which the ERR/Gy from high-dose fractionated exposure has been reported as much lower than that in acutely exposed survivors of the atomic bombs in Japan.^14^ In the Israel tinea capitis cohort (mean age at exposure of 7.8 years, doses from 1 to 6 Gy mostly in a single exposure) the ERR/Gy was higher, estimated at 5.01 (95% CI, 2.66-9.80).^15^ A Dutch study among survivors of childhood cancer reported an ERR/Gy of 0.30 (95% CI, 0.03-unknown) based on 97 meningioma cases; however, the study was based on prescribed cranial radiotherapy dose rather than meningioma location-specific dose and is therefore not directly comparable.^8^

We found that the dose-response association was approximately 4 times greater for exposure before age 10 years than after age 10 years and that it remained significantly elevated even 30 years after exposure (although there was limited power to distinguish between times scales of age at and time since exposure, which were correlated). Neither the study among survivors of the atomic bombs^13^ nor the tinea capitis study,^15^ nor the Dutch study among survivors of childhood cancer^8^ found that the radiation dose-response association decreased with increasing age at exposure. The French study^7^ among survivors of childhood cancer included in this analysis was the only prior study that reported a significant decreasing trend of the radiation dose-response association with increasing age at exposure. Understanding of meningeal tissue biology over the human life course is still evolving,^16^ and more research is needed both in populations and in vitro to confirm or potentially explain our results.

The influence of chemotherapy on meningioma risk is an area of much debate, given previous inconsistent findings. A potential dose-dependent risk associated with intrathecal methotrexate was suggested in the British study among survivors of childhood cancer,^5^ and recent analyses of the French nested case-control study found an OR of 3.01 (95% CI, 0.81-11.46) for the association between meningioma and systemic methotrexate.^7^ In this pooled study, although receipt of any methotrexate was associated with increased meningioma risk, there was no evidence of increasing risk with increasing dose of either systemic or intrathecal methotrexate. When a composite methotrexate score based on exposure tertiles for each route of administration was used, there was no evidence of a dose-response association.

A potential carcinogenicity of methotrexate has been suggested. Observational studies and reanalysis of randomized clinical trials of low-dose methotrexate for noncancer indications have reported an association with skin cancer and melanoma specifically but have not found a dose-response association.^17,18,19^ An association with lymphoma in patients with rheumatoid arthritis has also been debated.^20^ In our study, potential residual confounding by first cancer subtype may have contributed to the lack of dose-response association if, for example, individuals who were more likely to receive high-dose methotrexate were more predisposed to meningioma through an unknown pathway. If individuals treated with high-dose methotrexate were less likely to live (due to disease severity) than individuals treated with lower doses or no methotrexate, this might have affected control selection and introduced a potential informative censoring bias. Further follow-up of these findings is warranted.

### Strengths

This pooled analysis of 4 case-control studies has several strengths. To our knowledge, we have the largest number of meningioma cases exposed to radiation studied to date, providing enhanced precision to characterize the radiation dose-response association. Long-term follow-up provided insight into patterns of risks among longer-term survivors and at older ages. This is especially relevant for meningioma, as it occurred 15 or more years following the first cancer diagnosis. Additionally, using doses reconstructed to the meningioma site, rather than total brain dose or prescribed dose, provides a dose metric more relevant to tumor onset.

### Limitations

The study has several limitations. First, the estimated association of meningioma with radiation dose could be influenced by surveillance for subsequent tumors if individuals who had received higher doses were monitored more closely despite a lack of current or historical official recommendations.^4^ Because we see higher risks among survivors of CNS cancer compared with survivors of leukemia (both potentially exposed to cranial radiotherapy and higher surveillance), underlying genetic susceptibility and its association with cranial radiation dose (through first cancer type) might also have influenced our findings. Second, there may be some error in reconstructed dose estimates, but as these are unlikely to be differential between cases and controls, they would have biased the dose-response association toward the null. Fourth, our study had no information on SNs before cancer incidence or control selection, which could have potentially introduced bias. This issue might be negligible, since in prospective cohorts, approximately 90% of survivors of childhood cancer did not develop any SN during the entire follow-up^3,12^; therefore, it is more likely that cases are the second tumor and controls are SN-free. Furthermore, complete and specific data on genetic predisposition syndromes were not available for all studies. Only the French^7^ and British^5^ studies reported information on genetic syndromes ascertained by medical records and by self-report questionnaires, respectively. Among the genetic syndromes associated with predisposition to CNS tumors, neurofibromatosis type 2 (NF2) is the most strongly associated with meningioma risk. The French study^7^ reported that 40 subsequent CNS tumors occurred among individuals with genetic syndromes but none were NF2. In the British study,^5^ 2 meningioma cases occurred among individuals with genetic syndromes, both of which were NF2.^21^ In both studies,^5,7^ presence of any genetic syndrome was not associated with a higher risk of meningioma and did not significantly change the radiation dose-response.^8,21^ While uncommon and therefore unlikely to have had a major impact on our risk estimates,^22^ more detailed and systematic measurement of predisposition syndromes could provide deeper insight into this question.

With respect to surveillance, the intensity of monitoring for brain tumors was not collected. The radiation dose-response association did not differ significantly by calendar year of follow-up, which could be indirect evidence that it was not modified by potentially higher surveillance in more recent years. However, collection of monitoring data would enable this to be included in future studies.

## Conclusions

The results of this pooled case-control study suggest that the meninges are among the most radiosensitive tissues,^14^ especially for children younger than 10 years. These results support the reductions in whole brain irradiation over recent decades and the use of radiotherapy approaches that limit exposure of healthy tissue in children.^23,24^ The persistence of elevated risk of meningioma for more than 30 years after cranial radiotherapy could help inform surveillance guidelines for those treated as young children.

## Notes

Supplement.
eMethods.
**eTable 1.** Contributing Study Characteristics **eTable 2.** Distribution of Patient Characteristics by Receipt of Methotrexate Among Controls **eTable 3.** Associations Between Radiotherapy Dose and Patient Characteristics Among Controls **eTable 4.** Tests for Departure from Linearity for Radiation Dose-Response **eTable 5.** Estimates of the EOR/Gy for the Individual Studies **eTable 6.** Influence Analysis and Excess Odds Ratio per Gray (EOR/Gy) for Meningioma Among Childhood Cancer Survivors Omitting 1 Study at a Time **eTable 7.** Influence Analysis and Excess Odds Ratio per Gray (EOR/Gy) for Meningioma Among Survivors of Childhood Cancer Omitting 1 First Cancer Type at a Time, Restricted to Age Younger Than 10 y at Exposure **eTable 8.** Percentage of Patients Undergoing Radiotherapy, Mean and Median Brain Doses, Age at First Cancer Diagnosis, and Follow-up Information According to Type of First Cancer **eTable 9.** Estimates of Modification to the Linear Component (β) of the Radiation Dose Association by Age and Type of First Cancer **eTable 10.** Estimates of Modification to the Linear Component (β) of the Radiation Dose Association by Age and Latency **eTable 11.** Influence analysis and OR for Meningioma by Methotrexate Score Among Survivors of Childhood Cancer Omitting 1 Study at a Time **eTable 12.** Odds Ratios for Subsequent Meningiomas Following Treatment for Childhood Cancer by Methotrexate Dose **eTable 13.** Odds Ratios for Subsequent Meningiomas Following Treatment for Childhood Cancer by Epipodophyllotoxins Dose
eReferences.
