# Supplementary material for: Pooled Analysis of Meningioma Risk Following Treatment for Childhood Cancer
Source: JAMA Oncol. 2022 Oct 6;8(12):1756–64. doi: 10.1001/jamaoncol.2022.4425 (PMC9539736; doi:10.1001/jamaoncol.2022.4425)
Supplement: Supplement. — eMethods. eTable 1. Contributing Study Characteristics eTable 2. Distribution of Patient Characteristics by Receipt of Methotrexate Among Controls eTable 3. Associations Between Radiotherapy Dose and Patient Characteristics Among Controls eTable 4. Tests for Departure from Linearity for Radiation Dose-Response eTable 5. Estimates of the EOR/Gy for the Individual Studies eTable 6. Influence Analysis and Excess Odds Ratio per Gray (EOR/Gy) for Meningioma Among Childhood Cancer Survivors Omitting 1 Study at a Time eTable 7. Influence Analysis and Excess Odds Ratio per Gray (EOR/Gy) for Meningioma Among Survivors of Childhood Cancer Omitting 1 First Cancer Type at a Time, Restricted to Age Younger Than 10 y at Exposure eTable 8. Percentage of Patients Undergoing Radiotherapy, Mean and Median Brain Doses, Age at First Cancer Diagnosis, and Follow-up Information According to Type of First Cancer eTable 9. Estimates of Modification to the Linear Component (β) of the Radiation Dose Association by Age and Type of First Cancer eTable 10. Estimates of Modification to the Linear Component (β) of the Radiation Dose Association by Age and Latency eTable 11. Influence analysis and OR for Meningioma by Methotrexate Score Among Survivors of Childhood Cancer Omitting 1 Study at a Time eTable 12. Odds Ratios for Subsequent Meningiomas Following Treatment for Childhood Cancer by Methotrexate Dose eTable 13. Odds Ratios for Subsequent Meningiomas Following Treatment for Childhood Cancer by Epipodophyllotoxins Dose eReferences. [file jamaoncol-e224425-s001.pdf]

Supplemental Online Content

Withrow DR, Anderson H, Armstrong GT, et al. Pooled analysis of meningioma risk following treatment for childhood cancer. *JAMA Oncol*. Published online October 6, 2022. doi:10.1001/jamaoncol.2022.4425

eMethods.
eTable 1. Contributing Study Characteristics
eTable 2. Distribution of Patient Characteristics by Receipt of Methotrexate Among Controls
eTable 3. Associations Between Radiotherapy Dose and Patient Characteristics Among Controls
eTable 4. Tests for Departure from Linearity for Radiation Dose-Response
eTable 5. Estimates of the EOR/Gy for the Individual Studies
eTable 6. Influence Analysis and Excess Odds Ratio per Gray (EOR/Gy) for Meningioma Among Childhood Cancer Survivors Omitting 1 Study at a Time
eTable 7. Influence Analysis and Excess Odds Ratio per Gray (EOR/Gy) for Meningioma Among Survivors of Childhood Cancer Omitting 1 First Cancer Type at a Time, Restricted to Age Younger Than 10 y at Exposure
eTable 8. Percentage of Patients Undergoing Radiotherapy, Mean and Median Brain Doses, Age at First Cancer Diagnosis, and Follow-up Information According to Type of First Cancer
eTable 9. Estimates of Modification to the Linear Component (β) of the Radiation Dose Association by Age and Type of First Cancer
eTable 10. Estimates of Modification to the Linear Component (β) of the Radiation Dose Association by Age and Latency
eTable 11. Influence analysis and OR for Meningioma by Methotrexate Score Among Survivors of Childhood Cancer Omitting 1 Study at a Time
eTable 12. Odds Ratios for Subsequent Meningiomas Following Treatment for Childhood Cancer by Methotrexate Dose
eTable 13. Odds Ratios for Subsequent Meningiomas Following Treatment for Childhood Cancer by Epipodophyllotoxins Dose
eReferences.

This supplemental material has been provided by the authors to give readers additional information about their work.

© 2022 Withrow DR, et al. *JAMA Oncology.*

eMethods.

United States & Canada Childhood Cancer Survivor Study (CCSS-USCan)

The CCSS-USCan is a multi-center cohort study of five-year survivors of childhood cancer treated prior to age 21 years at 31 institutions in the U.S. and Canada. Our pooled study used data from a case-control study nested within the original cohort, which contains patients treated for first cancers between 1970 and 1986[1]. Unlike for the other studies, survivors of retinoblastoma were not eligible for inclusion in the CCSS-USCan. For each patient, doses were summed from all radiation treatment over the 10 years from the first cancer diagnosis. Follow-up for subsequent tumors occurred between 1975 and 2001. Subsequent primary tumors were identified through self-reported questionnaires and confirmed with pathology reports, medical records, or death certificates when available. Cases were matched to controls in a 1 to 4 ratio using age at diagnosis, sex, and duration of follow-up as matching factors. The CCSS-USCan contributed 66 cases and 264 controls. One case and 14 controls were subsequently excluded because they had unknown radiation status. Among 160 controls with non-zero doses of radiation to the meningioma location, the median dose was 6.9 Gy (range=0.01 to 50.4 Gy).

All available records for each case patient were reviewed by a pediatric oncologist (J. Neglia) to determine as precisely as possible the location of the subsequent CNS neoplasm. The radiation therapy records for all cases and control subjects were reviewed and abstracted (dates of therapy, beam energy, field size, field location, and total dose to each field (prophylactically or as treatment of recognized disease). Individual reconstructed radiation dose to the subsequent tumor location and the same location for the matched controls was based on radiotherapy information, age of the child at the time of therapy, and water phantom to estimate organ doses in a three-dimensional mathematical phantom that simulates a patient of any size. This method has been used to estimate organ doses for individual patients in several large studies of the long-term effects of radiation therapy and the overall approach is detailed elsewhere[2].

French Childhood Cancer Survivors Study (CCSS-FR)

The CCSS-Fr is a cohort of three-year survivors of non-leukemia childhood cancer survivors diagnosed between 1945 and 2000 and treated prior to age 18 years at five centers in France[3]. Cases of subsequent cancers were ascertained until 2016 using self-administered questionnaires, medical records, long-term follow-up consultation reports, causes of death from the national registry of death, and the French national health insurance reimbursement database. Cases were matched to controls in a 1 to 4 ratio based on age at diagnosis, sex and date of birth. The CCSS-FR contributed 86 cases and 340 controls. Among 202 controls with non-zero doses of radiation to the meningioma location, the median dose was 0.4 Gy (range 0.1 to 56.4 Gy).

Calculation of absorbed dose at the site of the subsequent tumor and the corresponding site in the controls was retrospectively reconstructed on the basis of the abstracted treatment information (machine, type of radiation, beam energy, irradiation technique, field size and shape, gantry and collimator angles, use of accessories, target volume location, and total delivered dose), mathematical gender- and age-specific phantoms modeling patient's anatomy in treatment position, and validated particle transport simulation models[4]. CNS subsequent tumor locations were delineated on phantoms based on radiological and medical records. When information was not retrieved on the subsequent tumor location (26% of cases), the mean dose to the cerebral lobe where the subsequent tumor occurred was considered. If this information was not recorded, the mean dose to the whole brain was used. If multiple SPN locations (e.g., diffuse meningioma) were reported on a given diagnosis date, we considered the mean dose to all locations.

British Childhood Cancer Survivors Study (BCCSS)

The BCCSS is a national, population-based cohort study of 17,980 five-year survivors of childhood cancer treated prior to age 15 years in the United Kingdom. Patients were diagnosed with their first cancer between 1942 and 1992. Demographic information as well as information on the first cancer and its treatment were obtained by self-report in a questionnaire, and through review of medical records. The survey asked patients to report if they had been diagnosed with any inherited conditions, including cancer predisposition syndromes. Follow-up for subsequent tumors occurred between 1947 and 2002 via linkage to the National Health Service Central Registers and by self-report among those who completed a follow-up questionnaire [5]. Second primary tumors were confirmed from histopathology reports and through writing to clinicians and pathologists. One-hundred and thirty-four meningioma cases were matched in a 1 to 1 ratio based on age at diagnosis, sex, and duration of follow-up. Twenty-five cases and controls were excluded due to unknown radiation status. Cases were not matched on first primary cancer type unless they were survivors of heritable retinoblastoma, in which case they were. Among 70 controls with non-zero doses of radiation to the meningioma location, the median dose was 11.3 Gy (range 0.01 to 48.5 Gy).

Detailed radiotherapy information for the first cancer was abstracted from medical records. Radiation dose to the subsequent tumor location and corresponding location in the matched controls were calculated using the dose calculation program Dos_Eg[6], which includes the construction of a patient phantom based on sex and height at the time of the treatment and contains 151 anatomical points of interest. The dose calculation used patient treatment parameters and the accurate positioning of the beams on the phantom and doses were calculated to 10 areas of the brain (cranium top, sella turcica, middle left brain hemisphere, middle right brain hemi-sphere, post-fossa, left lateral part of left brain hemisphere, right lateral part of right brain hemisphere, centre frontal part of left brain hemisphere, centre frontal part of right brain hemisphere, and glabella). The radiation dose at the site of

© 2022 Withrow DR, et al. *JAMA Oncology.*

the subsequent tumour for each case and corresponding location in the matched control was then estimated as that received in the particular area of the brain (sometimes the average of dose to 2 or 3 adjacent areas) in which the brain tumour arose.

Nordic Countries Childhood Cancer Survivor Study (CCSS-Nordic)

The data from CCSS-Nordic was drawn from a case-control study nested within a cohort of 25,120 childhood cancer survivors identified through the five Nordic population-based cancer registries (Denmark, Finland, Norway, Iceland, Sweden) [7]. Patients in the cohort were five-year survivors diagnosed with first cancer prior to age 20 years between 1957 and 1987. Follow-up spanned 1962 to 1991 and used linkage to cancer registries to identify cases. Thirteen meningioma cases were matched to 39 controls based on age at diagnosis, sex, duration of follow-up and calendar year of diagnosis. Among the controls, 13 had non-zero doses of radiation exposure to the tumor location, with a median of 0.84 Gy (range 0.01 to 54.7 Gy).

The calculation of absorbed dose is based on an algorithm that includes the primary beam and takes account of scatter in- and outside of the treatment beams and of leakage and scattered radiation around different treatment machines (derived from measurements around 28 different treatment machines in eight radiotherapy centers). Wedges and blocks can be simulated and correction for air in the lungs of the phantom is made. The absorbed dose was determined at 151 points in the body for each course of radiation therapy for each child. All dose values are valid for water. The absorbed dose received at the site of SMST for the case was compared with the dose at the corresponding site in the controls.

© 2022 Withrow DR, et al. *JAMA Oncology.*

eTABLES

eTable 1. Contributing Study Characteristics

Study	Region(s)	First cancer diagnosis years	Follow-up years	Matching factors	Cases/ Controls[a]	Median radiation dose[b] (range), Gy
CCSS-USCan[7]	US, Canada	1970-1986	1975-2001	Age at diagnosis (±2 years), sex, and duration of follow-up	66/264	6.9 (0.01 to 50.4)
CCSS-FR[8 c]	France	1945-2000	1948-2016	Age at diagnosis (±2 years), sex, calendar year of first cancer diagnosis (±5 years), duration of follow-up	86/340	0.4 (0.1 to 56.4)
BCCSS[6]	UK	1942-1992	1947-2002	Age at diagnosis (in three year age bands), sex, duration of follow-up	134/134	11.3 (0.01-48.5)
CCSS-Nordic[10]	Denmark, Finland, Norway, Iceland, Sweden	1957-1987	1962-1991	Age at diagnosis, sex, duration of follow-up and calendar year of diagnosis (±3 years)	13/39	0.84 (0.01 to 54.7)

[a] Includes all cases and controls from original studies. Study subjects with unknown radiation status were subsequently excluded from further analyses (1 case and 14 controls in CCSS-USCan & 25 cases and controls in BCCSS). [b] Radiation dose to the tumor site location among irradiated controls. [c] CCSS-FR did not include leukemia patients. For more information see Supplemental Methods. CCSS: Childhood Cancer Survivor Study

© 2022 Withrow DR, et al. *JAMA Oncology.*

eTable 2. Distribution of Patient Characteristics by Receipt of Methotrexate Among Controls

	Any methotrexate [a]				Intrathecal methotrexate [b]				Systemic methotrexate [b]			
	No	%	Yes	%	No	%	Yes	%	No	%	Yes	%
Total [a]	548	77.7	157	22.3	556	83.5	110	16.5	575	86.3	91	13.7
First cancer type												
Leukemia	17	15.5	93	84.5	22	20.8	84	79.2	65	61.3	41	38.7
CNS cancer	101	98.1	2	1.9	91	100.0	0	0.0	89	97.8	2	2.2
Hodgkin lymphoma	49	100.0	0	0.0	46	100.0	0	0.0	46	100.0	0	0.0
Non-Hodgkin lymphoma (NHL)	12	34.3	23	65.7	17	50.0	17	50.0	21	61.8	13	38.2
Kidney (Wilms tumor)	121	100.0	0	0.0	119	100.0	0	0.0	119	100.0	0	0.0
Neuroblastoma	71	100.0	0	0.0	70	100.0	0	0.0	70	100.0	0	0.0
Soft tissue sarcoma	65	94.2	4	5.8	67	100.0	0	0.0	63	94.0	4	6.0
Bone cancer	33	64.7	18	35.3	50	100.0	0	0.0	33	66.0	17	34.0
Retinoblastoma	23	100.0	0	0.0	22	100.0	0	0.0	22	100.0	0	0.0
Other	56	76.7	17	23.3	52	85.2	9	14.8	47	77.0	14	23.0
Age at diagnosis, years												
0-4	255	78.7	69	21.3	259	81.4	59	18.6	281	88.4	37	11.6
5-9	154	74.0	54	26.0	166	82.6	35	17.4	174	86.6	27	13.4
10-14	98	80.3	24	19.7	107	89.2	13	10.8	101	84.2	19	15.8
15-20	41	80.4	10	19.6	24	88.9	3	11.1	19	70.4	8	29.6
Year of first cancer diagnosis												
1942-1960	31	100.0	0	0.0	29	100.0	0	0.0	29	100.0	0	0.0
1960-1969	85	95.5	4	4.5	75	100.0	0	0.0	73	97.3	2	2.7
1970-1979	276	73.8	98	26.2	281	78.7	76	21.3	308	86.3	49	13.7
1980-2000	156	73.9	55	26.1	171	83.4	34	16.6	165	80.5	40	19.5
Brain dose (Gy)												
0	200	76.9	60	23.1	208	88.9	26	11.1	188	80.3	46	18.7
>0-<20	274	91	27	9	271	93.1	20	6.9	279	95.9	12	4.2
20-<40	39	40.2	58	59.8	42	43.8	54	56.3	67	69.8	29	42.6
≥40	16	76.2	5	23.8	16	84.2	3	15.8	16	84.2	3	15.8
Median Brain dose (IQR), Gy [c]	0.3 (0.1-6.1)		24 (18-24.3)		0.3 (0.1-7.2)		24 (20.5-24.3)		0.5 (0.1-20.5)		24 (18.0-24.1)	

[a] Excluded 38 patients with unknown methotrexate. [b] Excluded 77 patients with unknown intrathecal and systemic methotrexate. 49 patients received both intrathecal and systemic methotrexate. [c] Among controls exposed to radiation.

© 2022 Withrow DR, et al. *JAMA Oncology.*

eTable 3. Associations Between Radiotherapy Dose and Patient Characteristics Among Controls

	Radiotherapy dose to brain						Any brain dose	
	0 Gy		0.1 to 20 Gy		>20 Gy			
	N	%	N	%	N	%	N	%
Total [a]	267	35	316	41	129	17	462	63
First cancer type								
Leukemia	20	17	18	16	70	60	88	76
Central nervous system	34	31	30	27	41	37	71	64
Hodgkin lymphoma	0	0	51	93	1	2	52	95
Non-Hodgkin lymphoma	13	35	13	35	10	27	23	62
Kidney (Wilms tumor)	23	18	101	81	0	0	101	81
Neuroblastoma	34	48	33	46	1	1	34	48
Soft tissue sarcoma	41	56	27	37	1	1	28	38
Bone cancer	36	68	15	28	2	4	17	32
Retinoblastoma	10	14	12	16	1	1	13	18
Other	56	243	16	70	2	9	18	78
Age at diagnosis, years								
0-4	121	36	148	44	57	17	205	61
5-9	64	29	98	45	52	24	150	68
10-14	57	45	51	40	15	12	66	52
15-20	25	47	19	36	5	9	24	45
Year of first cancer diagnosis								
1942-1959	13	42	18	58	0	0	18	58
1960-1969	35	36	56	58	6	6	62	64
1970-1979	113	29	164	42	93	24	257	66
1980-2000	106	50	78	37	30	14	108	51
Chemotherapy								
No	100	46	87	40	25	11	112	51
Yes	160	33	213	44	93	19	306	63
Unknown	7	21	16	47	11	32	27	79
Chemotherapy agents								
Alkylating agents	116	29	121	30	53	13	174	44
Anthracyclines	90	47	70	36	26	13	96	50
Epipodophyllotoxins	17	49	12	34	5	14	17	49
Platinum compounds	22	54	13	32	4	10	17	41
Antimetabolites	51	31	31	19	74	45	105	64
IT Methotrexate	26	24	20	18	57	52	77	70
Systemic Methotrexate	46	51	12	13	32	35	44	48

[a] May not add to total due to subjects with unknown dose.

© 2022 Withrow DR, et al. *JAMA Oncology.*

eTable 4. Tests for Departure from Linearity for Radiation Dose-Response

Model #	Model Description[a]	Parms[b]	Deviance[c]	AIC[d]	Referent model	p[e]
1	Baseline	0	420.09	428.09		
**2**	**Linear**	**1**	**287.43**	**297.43[f]**	**1**	**<0.001**
3	Quadratic	1	299.89	308.89	1	<0.001
4	Linear quadratic	2	287.42	299.42	2	0.90
5	Linear-exponential (linear)	2	287.42	299.42	2	0.90
6	Linear-exponential (quadratic)	2	287.42	299.42	2	0.90
7	Linear-quadratic-exponential (linear)	3	287.41	301.41	4	0.92

[a] Models for radiation dose, d, include: linear: $EOR(d) = \beta_1 d$; quadratic: $EOR(d)=\beta_2 d^2$; linear quadratic: $EOR(d)= \beta_1 d + \beta_2 d^2$; linear-exponential (linear): $EOR(d) = \beta_1 d \exp(\beta_3 d)$; linear-exponential (quadratic): $EOR(d) = \beta_1 d \exp(\beta_4 d^2)$; and linear-quadratic-exponential (linear): $EOR(d) = (\beta_1 d + \beta_2 d^2) \exp(\beta_3 d)$, where β_1 and β_2 defines the linear slope parameter in excess risk per Gray (Gy) and β_3 and/or β_4 defines the curvature. Background risk adjusted for type of first cancer (leukemia, CNS cancer, other cancers) and methotrexate (yes/np). [b] Number of dose parameters in the model. [c] Model deviance. [d] AIC, Akaike Information Criterion. A smaller value denotes a more preferred model. [e] p-value for no improvement in model fit relative to the referent model number. [f] The linear model, $EOR(d) = \beta_1 d$ was the preferred model (in bold) based on the lowest AIC. This model corresponds to a straight-line dose-response relation, in which β1 equals the slope (i.e., EOR per Gy).

eTable 5. Estimates of the EOR/Gy for the Individual Studies

**Study**	**EOR/Gy (95% CI)**	**P[a]**	**P[b]**	**P[c]**
CCSS-USCan [d]	1.07 (0.15-7.9)	<0.01	0.37	0.84
CCSS-Fr [e]	1.55 (0.43-5.65)	<0.01	0.37	
BCCSS-UK [f]	4.89 (0.39-59.89)	<0.01	0.60	
CCSS-Nordic [g]	0.89 (0.07-10.54)	<0.01	0.80	

[a] P-value for linear trend with radiation dose. [b] P-value for test of no departure from linearity using a log linear term. [c] P-value for test of heterogeneity across studies. [d] Estimates based on conditional logistic regression model matched on sex, age at first cancer diagnosis and duration of follow-up. Further adjustment for type of first cancer (leukemia, CNS cancer, other cancers). [e] Estimates based on conditional logistic regression model matched on sex, age at first cancer, duration of follow-up and calendar year of first cancer diagnosis. Further adjustment for type of first cancer (CNS vs other cancers). [f] Estimates based on conditional logistic regression model matched on sex, age at first cancer and duration of follow-up. [g] Estimates based on conditional logistic regression model matched on sex, age at first cancer, duration of follow-up and calendar year of first cancer diagnosis. EOR/Gy: Excess odds ratio/Gray; CI: Confidence interval.

© 2022 Withrow DR, et al. *JAMA Oncology.*

eTable 6. Influence Analysis and Excess Odds Ratio per Gray (EOR/Gy) for Meningioma Among Childhood Cancer Survivors Omitting 1 Study at a Time

	**EOR/Gy[a]**
Overall	1.44 (0.58, 3.57)
Omitting CCSS-USCan	1.95 (0.66, 5.81)
Omitting CCSS-Fr	1.42 (0.39, 5.49)
Omitting BCCSS-UK	1.20 (0.46, 3.17)
Omitting CCSS-Nordic	1.58 (0.57, 4.33)

[a] EOR model included adjustment for type of first cancer (others, leukemia and CNS) and for methotrexate (y/n).

eTable 7. Influence Analysis and Excess Odds Ratio per Gray (EOR/Gy) for Meningioma Among Survivors of Childhood Cancer Omitting 1 First Cancer Type at a Time, Restricted to Age Younger Than 10 y at Exposure

	**EOR/Gy[a]**
Overall	1.58 (0.58, 4.33)
Omitting leukemia	1.45 (0.53, 4.01)
Omitting CNS	2.63 (0.58, 11.80)
Omitting Hodgkin	1.90 (0.67, 5.32)
Omitting NHL	1.23 (0.47, 3.23)
Omitting Kidney (Willm's tumor)	1.05 (0.43, 2.57)
Omitting Neuroblastoma	1.25 (0.50, 3.17)
Omitting soft tissue sarcoma	1.13 (0.45, 2.83)
Omitting bone cancer	1.93 (0.70, 5.31)
Omitting retinoblastoma	1.48 (0.58, 3.79)
Omitting other	1.14 (0.43, 3.06)

[a] OR model included adjustment for type of first cancer (others, leukemia and CNS) and methotrexate (y/n).

© 2022 Withrow DR, et al. *JAMA Oncology.*

eTable 8. Percentage of Patients Undergoing Radiotherapy, Mean and Median Brain Doses, Age at First Cancer Diagnosis, and Follow-up Information According to Type of First Cancer

First cancer type	Cases	Controls	Any radiotherapy (%)[a]	Brain dose (Gy)[b]		Age at 1st cancer diagnosis[a]	Age at Meningioma diagnosis[c]	Time to diagnosis of meningioma (years) [c]
	n	n		Mean	Median (IQR)	Median (IQR)	Median (IQR)	Median (IQR)
Leukemia	68	116	83	23.6	24.0 (21.1-24.2)	4 (3-7)	25 (20-27)	19 (16-22)
CNS	153	111	69	24.7	27.0 (5.3-38.8)	6 (4-10)	29 (22-35)	21 (14-27)
HL	9	55	100	1.4	0.6 (0.3-1.2)	12 (8-15)	37 (27-43)	27 (15-30)
NHL	9	37	68	11.4	2.5 (0.1-24.1)	7 (5-13)	25 (16-29)	18 (10-23)
Kidney	3	125	86	0.6	0.1 (0.1-0.2)	4 (2-5)	33 (33-39)	31 (30-36)
Neuroblastoma	4	71	62	1.1	0.1 (0.04-0.3)	1 (0-2)	29 (19-32)	24 (15-30)
Soft tissue sarcoma	6	73	63	1.2	0.1 (0-0.4)	7 (3-10)	33 (15-47)	29 (12-43)
Bone cancer	2	53	43	3.2	0.02 (0-0.3)	10 (8-12	30 (29-31)	14 (9-20)
Retinoblastoma	5	74	56	6.2	2.2 (1.0-8.1)	1 (0-3)	33 (32-35)	31 (31-33)
Others	14	23	30	3.9	0.2 (0.1-2.0)	9 (5-12)	35 (26-38)	24 (21-34)
All cancers	273	738	69	9.5	0.6 (0.1-21.9)	5 (3-9)	27 (21-34)	21 (15-27)

[a] Among controls. [b] Among controls with known radiation dose. [c] Among cases.

© 2022 Withrow DR, et al. *JAMA Oncology.*

eTable 9. Estimates of Modification to the Linear Component (β) of the Radiation Dose Association by Age and Type of First Cancer[a]

	Cases/ Controls	EOR/Gy	95% CI
Age at exposure (yrs) & type of first primary cancer			
<5, non-CNS	61/295	2.62	(0.83, 9.44)
5-9, non-CNS	40/171	3.51	(1.10, 12.28)
≥10, non-CNS	14/141	0.98	(0.19, 4.57)
<5, CNS	52/31	1.13	(0.25, 5.82)
5-9, CNS	52/43	1.13	(0.28-5.11)
≥10, CNS	46/31	0.32	(0.07-1.46)
p		0.22	

[a] EOR model included adjustment for type of first cancer (others, leukemia and CNS) and methotrexate (yes/no). EOR/Gy: Excess odds ratio/Gray; CI: confidence interval; CNS: central nervous system.

eTable 10. Estimates of Modification to the Linear Component (β) of the Radiation Dose Association by Age and Latency[a]

	Cases/ Controls	EOR/Gy	95% CI
Age at exposure (yrs) & latency (yrs)			
<5, latency <20	52/150	1.93	(0.45, 11.69)
5-9, latency <20	32/79	0.83	(0.19, 4.22)
≥10, latency <20	33/89	0.42	(0.09, 2.36)
<5, latency 20+	61/176	2.20	(0.57, 10.61)
5-9, latency 20+	60/135	5.02	(1.37, 22.85)
≥10, latency 20+	27/83	0.68	(0.13, 3.73)
p		0.06	

[a] EOR model included adjustment for type of first cancer (others, leukemia and CNS), epipodophyllotoxin (y/n) and methotrexate (y/n). EOR/Gy: Excess odds ratio/Gray; CI: confidence interval; CNS: central nervous system.

© 2022 Withrow DR, et al. *JAMA Oncology.*

eTable 11. Influence analysis and OR[a] for Meningioma by Methotrexate Score Among Survivors of Childhood Cancer Omitting 1 Study at a Time

	Methotrexate Score [b]						
	Not exposed	1	2	3	4+	p-trend	p-trend[c]
All studies	1.0	2.79 (0.75, 10.40)	3.65 (1.38, 9.63)	3.68 (1.43, 9.48)	4.48 (1.48, 13.61)	0.004	0.47
Omitting CCSS-US	1.0	1.59 (0.23,11.11)	2.82 (0.65, 12.13)	2.85 (0.77, 10.55)	6.43 (1.49, 27.72)	0.018	0.55
Omitting CCSS-FR	1.0	5.01 (0.79, 31.58)	8.04 (2.12, 30.57)	6.75 (1.76, 25.95)	8.98 (2.00, 40.13)	0.004	0.97
Omitting BCCSS	1.0	3.13 (0.81, 12.16)	2.60 (0.91, 7.37)	3.24 (1.18, 8.91)	2.38 (0.62, 9.19)	0.070	0.11
Omitting CCSS-Nordic	1.0	2.56 (0.68, 9.52)	3.34 (1.26, 8.85)	3.39 (1.31, 8.79)	4.04 (1.32, 12.32)	0.008	0.47

[a] Adjusted for type of first cancer (other, leukemia & CNS) and radiation dose categories.

[b] Intrathecal and systemic methotrexate dose distributions among controls were divided into tertiles. Each study subject was assigned a score of 0, 1, 2, or 3 for each route of administration, depending on whether the subject received none or fell into the lower, middle, or upper third of the distribution, respectively. The scores of the intrathecal and systemic methotrexate were then summed for each study subject in order to obtain a "methotrexate score", which ranged from 0 to 6. Patients with missing intrathecal or systemic methotrexate dose were excluded from this analysis. Totals do not sum to study totals due to subjects with unknown methotrexate receipt.

[c] Among patients exposed to any methotrexate

© 2022 Withrow DR, et al. *JAMA Oncology.*

eTable 12. Odds Ratios for Subsequent Meningiomas Following Treatment for Childhood Cancer by Methotrexate Dose

Characteristics	Cases	Controls	OR[a] (95% CI)
**Intrathecal methotrexate dose, mg/m^2 (mean)**			
0	184	591	1
0.001-57.1 (39.4)	26	24	5.26 (1.84, 15.03)
57.2-75.0 (65.1)	16	25	4.27 (1.41, 12.88)
75.1-160.0 (111.9)	12	28	2.17 (0.73, 6.39)
160.1-1302 (303.0)	11	26	2.54 (0.72, 8.93)
p-trend			0.41
p-trend among exposed			0.002
**Systemic methotrexate dose, mg/m^2 (mean)**			
0	202	617	1.0
0.0-1043.1 (504.1)	20	22	2.89 (1.04, 8.04)
1043.2-3490.7 (1952.6)	23	22	2.24 (0.92, 5.42)
3490.8-19170.0 (9017.3)	4	22	1.25 (0.28, 5.63)
19170.1-375,000 (101,552)	5	22	6.63 (1.08, 40.92)
p-trend			0.35
p-trend among exposed			ne

[a] Adjusted for type of first cancer (other, leukemia & CNS) and radiation dose categories.

eTable 13. Odds Ratios for Subsequent Meningiomas Following Treatment for Childhood Cancer by Epipodophyllotoxins Dose

Characteristics	Cases	Controls	OR (95% CI)
**Epipodophyllotoxins dose, mg/m^2 (mean)**			
0	234	670	1
0.1-883.3 (558.3)	4	11	2.02 (0.36, 11.39)
883.4-1602.1 (1194.8)	6	12	4.03 (0.66, 24.4)
1602.2-51640 (6437.9)	2	11	0.33 (0.02, 4.70)
Missing/Unknown	27	34	2.55 (0.78, 8.24)
p-trend			0.49
p-trend among exposed			0.25

[a] ORs computed using conditional logistic regression with adjustment for matching factors, radiation dose categories, methotrexate and type of first cancer.

[b] Based on tertiles of dose distribution among controls who received epipodophyllotoxins.

© 2022 Withrow DR, et al. *JAMA Oncology.*

eReferences

1. Neglia JP, Robison LL, Stovall M, et al. New primary neoplasms of the central nervous system in survivors of childhood cancer: a report from the Childhood Cancer Survivor Study. *J Natl Cancer Inst.* 2006;98(21):1528-1537.
2. Stovall M, Donaldson SS, Weathers RE, et al. Genetic effects of radiotherapy for childhood cancer: gonadal dose reconstruction. *International Journal of Radiation Oncology* Biology* Physics.* 2004;60(2):542-552.
3. de Vathaire F, Hawkins M, Campbell S, et al. Second malignant neoplasms after a first cancer in childhood: temporal pattern of risk according to type of treatment. *Br J Cancer.* 1999;79(11-12):1884-1893.
4. Veres C, Allodji RS, Llanas D, et al. Retrospective reconstructions of active bone marrow dose-volume histograms. *International Journal of Radiation Oncology* Biology* Physics.* 2014;90(5):1216-1224.
5. Taylor AJ, Little MP, Winter DL, et al. Population-based risks of CNS tumors in survivors of childhood cancer: the British Childhood Cancer Survivor Study. *J Clin Oncol.* 2010;28(36):5287-5293.
6. Diallo I, Lamon A, Shamsaldin A, Grimaud E, De Vathaire F, Chavaudra J. Estimation of the radiation dose delivered to any point outside the target volume per patient treated with external beam radiotherapy. *Radiotherapy and oncology.* 1996;38(3):269-271.
7. Svahn-Tapper G, Garwicz S, Anderson H, et al. Radiation dose and relapse are predictors for development of second malignant solid tumors after cancer in childhood and adolescence: a population-based case-control study in the five Nordic countries. *Acta Oncol.* 2006;45(4):438-448.

© 2022 Withrow DR, et al. *JAMA Oncology.*
